# Development and Validation of a 20K Single Nucleotide Polymorphism (SNP) Whole Genome Genotyping Array for Apple (*Malus × domestica* Borkh)

**DOI:** 10.1371/journal.pone.0110377

**Published:** 2014-10-10

**Authors:** Luca Bianco, Alessandro Cestaro, Daniel James Sargent, Elisa Banchi, Sophia Derdak, Mario Di Guardo, Silvio Salvi, Johannes Jansen, Roberto Viola, Ivo Gut, Francois Laurens, David Chagné, Riccardo Velasco, Eric van de Weg, Michela Troggio

**Affiliations:** 1 Research and Innovation Centre, Fondazione Edmund Mach, San Michele all’Adige, Trento, Italy; 2 CNAG – Centro Nacional de Análisis Genómico, Parc Científic de Barcelona, Barcelona, Spain; 3 Wageningen UR Plant Breeding, Wageningen University and Research Centre, Wageningen, The Netherlands; 4 DipSA - University of Bologna, Bologna, Italy; 5 Biometris, Wageningen University and Research Centre, Wageningen, The Netherlands; 6 INRA, UMR1345 Institut de Recherche en Horticulture and Semences, Beaucouzé, France; 7 Plant & Food Research, Palmerston North Research Centre, Palmerston North, New Zealand; Oregon State University, United States of America

## Abstract

High-density SNP arrays for genome-wide assessment of allelic variation have made high resolution genetic characterization of crop germplasm feasible. A medium density array for apple, the IRSC 8****K SNP array, has been successfully developed and used for screens of bi-parental populations. However, the number of robust and well-distributed markers contained on this array was not sufficient to perform genome-wide association analyses in wider germplasm sets, or Pedigree-Based Analysis at high precision, because of rapid decay of linkage disequilibrium. We describe the development of an Illumina Infinium array targeting 20****K SNPs. The SNPs were predicted from re-sequencing data derived from the genomes of 13 *Malus × domestica* apple cultivars and one accession belonging to a crab apple species (*M. micromalus*). A pipeline for SNP selection was devised that avoided the pitfalls associated with the inclusion of paralogous sequence variants, supported the construction of robust multi-allelic SNP haploblocks and selected up to 11 entries within narrow genomic regions of ±5 kb, termed focal points (FPs). Broad genome coverage was attained by placing FPs at 1 cM intervals on a consensus genetic map, complementing them with FPs to enrich the ends of each of the chromosomes, and by bridging physical intervals greater than 400 Kbps. The selection also included ∼3.7****K validated SNPs from the IRSC 8****K array. The array has already been used in other studies where ∼15.8****K SNP markers were mapped with an average of ∼6.8****K SNPs per full-sib family. The newly developed array with its high density of polymorphic validated SNPs is expected to be of great utility for Pedigree-Based Analysis and Genomic Selection. It will also be a valuable tool to help dissect the genetic mechanisms controlling important fruit quality traits, and to aid the identification of marker-trait associations suitable for the application of Marker Assisted Selection in apple breeding programs.

## Introduction

Cultivated apple (*Malus × domestica*) is the most economically important deciduous fruit tree crop worldwide [1]. Breeding of novel cultivars with superior fruit quality characteristics is a slow and costly process, because of the extended juvenility period of the species. Typically, the selection process takes on average more than 20 years from seed to introduction, even when rootstocks conferring precocious flowering are exploited. Breeding for disease resistance takes even longer, requiring introgression of novel resistance genes from wild germplasm, followed by successive generations of back-crossing to restore fruit quality to a commercially acceptable level. The use of molecular markers could accelerate and enhance the breeding process, particularly for traits that are difficult to select for phenotypically, such as pyramided disease resistances, or for traits that are expressed only in mature trees, such as fruit characteristics.

Recent advances in genomics technologies have enabled the sequencing of the ‘Golden Delicious’ genome [2] and the subsequent development of a whole genome genotyping (WGG) (micro-) array for the species [3]. The International RosBREED Single nucleotide polymorphism (SNP) Consortium (IRSC) WGG array contains 7,867 *Malus* SNP markers as well as 921 SNPs derived from *Pyrus* [4]. The IRSC array greatly facilitated the development of high density linkage maps for segregating apple progenies [5]–[7]. It was used in a Genome-Wide Association Study (GWAS) of the genetic control of several significant fruit traits [8], for the implementation of Genomic Selection (GS) [9] and for resolving pedigrees [10]. However, these 8,788 potential genetic markers are not sufficient to perform GWAS in wider germplasm sets, or to perform Pedigree-Based Analysis (PBA) [11] with high levels of precision. This is due to the rapid linkage disequilibrium decay in apple [12], the limited proportion of robust, easy to score makers included on the IRSC SNP array [6], [10], and their uneven distribution across the genome. A higher density array, with robust genome-wide markers is therefore required to perform such studies successfully.

In this investigation, we describe the development of such a high density WGG array for apple, using a focal point approach and stringent selection criteria built from experience of the analysis of the apple IRSC array [3], [5], [6]. A haplotype-targeting strategy similar to that adopted to design the IRSC array was implemented for the design of the array to combine information from individual SNPs into haploblocks and provide fully informative multi-allelic markers. We also summarize available metadata on the application of the array to a separate genetic mapping study.

## Materials and Methods

### SNP Discovery Panel and Re-sequencing

To enable the identification of SNPs, a discovery panel comprising the following 13 apple cultivars, including some of the core European apple breeding founder varieties [13]–[15], was re-sequenced using short-read sequencing technology: ‘Braeburn’, ‘Cox’s Orange Pippin’, ‘Common Antonovka’, ‘Delicious’, ‘Dr. Oldenburg’, F2-26829-2-2, ‘Fuji’, ‘Jonathan’, ‘Lady Williams’, ‘McIntosh’, ‘Macoun’, ‘Priscilla-NL’ and ‘Worcester Pearmain-USA’. Additionally, a scab-resistant accession of *M. micromalus* and two *M. × domestica* double haploid (DH) accessions, X9273 and X9748, which were derived from ‘Golden Delicious’, were included [16]. Leaf material was procured from various institutions (Table 1). For ‘Priscilla-NL’ and ‘Worcester Pearmain-USA’, the country of origin of the leaf material is included, to distinguish them from other genotypes with the same cultivar name [17]. The DHs were included to help identify pseudo-SNPs created from paralogous sequences of the apple genome that are erroneously assembled into a single locus, or that are located at different segments/chromosomes but that are targeted by the same Illumina probes. Since the DH lines are homozygous across their entire genomes, any heterozygous calls in these genotypes were thus considered evidence of paralogous sequences. DNA was extracted from freeze-dried, newly emerged leaf material using a phenol-chloroform isoamyl alcohol extraction method [10] and quantified with a Qubit fluorometer (Life Technologies). Sequencing libraries were constructed according to the TruSeq DNA sample preparation protocol (Illumina) with minor modifications, in particular employing double size selection steps. Two micrograms of genomic DNA were fragmented with a Covaris E210 and size selected to 300–600 bps. The resulting fragments were end-repaired, adenylated and ligated to Illumina paired-end adaptors. The size of the library was confirmed on the BioAnalyzer 2100 (Agilent) and the library was sequenced on an Illumina HiSeq 2000 platform with paired end runs of 2×101+7 bps. Base calling and quality control were performed using the Illumina RTA sequence analysis pipeline.

**Table 1:** Origin of samples and re-sequencing statistics for the 14 genotypes of the discovery panel and the two double haploids.

Sample name	Source of leafmaterial*	Tot readpairs	Mean percentunique reads	Meancoverage
‘Dr Oldenburg’	JKI	134,605,846	55.98	43.13
‘Fuji’	JKI	116,541,162	56.82	37.95
‘Lady Williams’	JKI	115,667,659	56.17	38.12
F2-26829-2-2	UNIBO	110,254,636	55.14	35.52
‘Macoun’	JKI	95,279,675	55.35	30.77
‘Cox’s OrangePippin’	WUR	98,094,673	56.53	32.61
‘Worchester Pearmain-USA’ PI 206035	PGRU-Geneva	104,600,260	54.67	34.30
‘Jonathan’	WUR	137,126,235	56.00	45.45
*Malus* *micromalus*	WUR	117,046,241	50.11	34.65
‘McIntosh’	INRA	135,946,435	51.10	44.33
‘Delicious’	INRA	139,815,864	57.49	45.88
‘Braeburn’	KUL	122,735,693	50.48	39.37
CommonAntonovka	VNIIISPK	133,423,835	50.73	43.32
‘Priscilla-NL’	WUR	127,017,433	55.02	41.17
X9748	INRA	197,011,579	59.17	66.84
X9273	INRA	207,626,734	58.81	69.70

The 16 genomes were sequenced on 8 Illumina HiSeq 2000 lanes. Double haploids (DHs, acc. X9748 and X9273) feature almost a double coverage compared with the other genomes; the total number of read pairs ranges from 95 Million to 207 Million. The percentage of uniquely aligning reads is evenly spread over each genome and ranges from 50 to 60% of the total. The mean coverage similarly ranges from 30 to 45X, while it is higher for the DHs.

*(JKI) Julius Kühn-Institut; (UNIBO) Università di Bologna; (WUR) Wageningen University and Research centre; (PGRU-Geneva) Plant Genetic Resources Unit; (INRA) Institut National de la Recherche Agronomique; (KUL) Katholieke Universiteit Leuven; (VNIIISPK) The All Russian Research Institute of Horticultural Breeding.

### Read Alignment, Variant Detection and Quality Filtering

**Figure 1:**
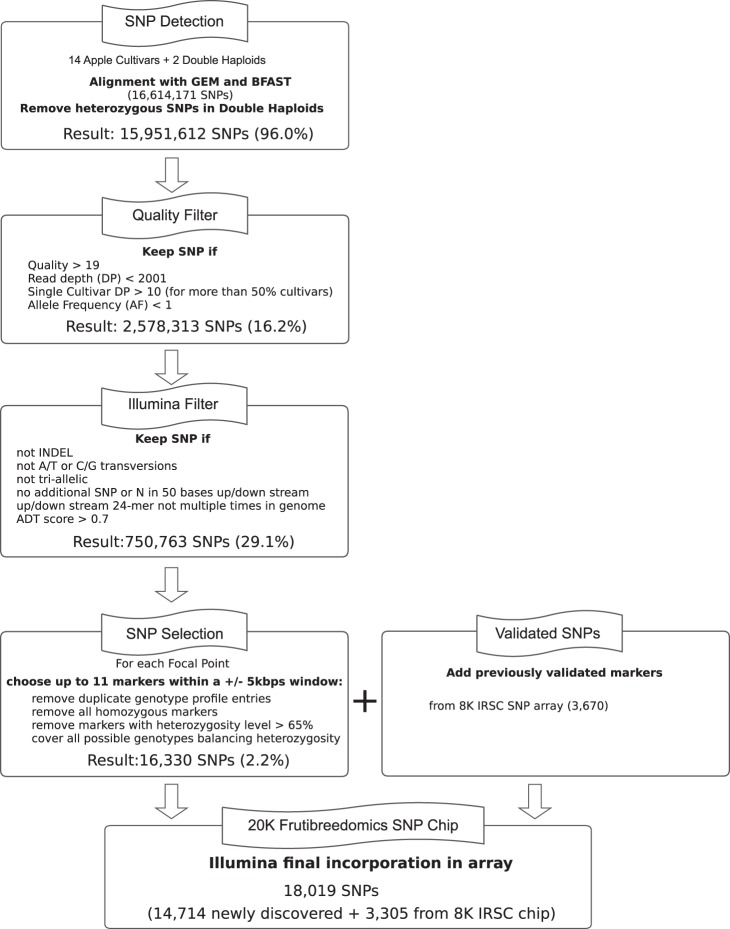
The SNP selection workflow. The SNP selection process was a cascade involving four steps: Detection, Quality Filtering, Illumina specific Filtering and SNP Selection, with the number of SNPs reduced after each step. The specific filtering criteria have been reported for each filtering stage, as well as the number of resulting SNPs, with the corresponding percentage survival, relative to the total number of SNP markers after the previous step. From the 20,000 selected, Illumina successfully incorporated a total of 18,019 SNP probes into the new array, 14,714 of which were newly discovered and 3,305 previously validated.

A schematic representation of all the steps in the pipeline for array development is given in Figure 1. As the first step, reads were sequentially aligned to the primary assembly and three alternative assemblies of the ‘Golden Delicious’ genome v2.0 (http://www.rosaceae.org/species/malus/malus_x_domestica/genome_v2.0) allowing up to seven mismatches in a four-step exhaustive alignment procedure using GEM [18] and BFAST [19]. Version 2.0 of the apple genome was created by removing 34,882 problematic contigs from the previous version [2]. The primary assembly, representing about 80% of the assembled and anchored genome, and the three alternative assemblies were produced following the NCBI AGP v2.0 specifications (http://www.ncbi.nlm.nih.gov/projects/genome/assembly/agp/AGP_Specification_v2.0.shtml).

Read pairs that always mapped to a single genomic location were kept in the alignment file for that reference at each step, while unaligned read pairs were submitted to the next mapping round. Pairs that did not align uniquely to any of the references were discarded (Figure 1). As a result, four .bam files were generated for each sample from the four alternative assemblies. Variant calling was performed for each of these .bam files separately, applying samtools and bcftools (version 0.1.18) [20] using default parameters. Positions with significant strand bias (p-value<0.001), significant tail distance bias (p-value<0.05) or within regions of low mapping confidence [21] were excluded. The variant-calling pipeline was set to produce genotype calls for each variant for all the re-sequenced cultivars. Calls with a support value of at least 10 reads were retained in the .vcf file, while the others were considered unreliable and therefore set to empty. To avoid the inclusion of potential paralogy-related SNPs, variants with a heterozygous genotype in the DHs were filtered out. Finally, known ambiguous bases in the primary reference were annotated.

A quality filter was then applied to remove potentially unreliable variants. Custom scripts written in Python (www.python.org) were developed to remove variants with low phred-scaled quality scores (i.e. below 20); a high combined read depth (i.e. higher than 2,000); and a low single-cultivar read depth (i.e. lower than 10) in more than 50% of the cultivars. A minimum phred-scaled quality score of 20 was chosen to ensure that only SNPs with a probability of less than 1% for the alternative allele being called wrongly were selected, and the maximum read depth value was used to ensure the removal of SNPs derived from paralogous regions rather than true heterozygous regions as done previously in the development of the IRSC apple array [3] and the 9****K peach array [22]. Additionally, a cut-off of at least ten reads per single cultivar in more than 50% of the cultivars was used to distinguish real variants from potential sequencing errors. The 50% cut-off was chosen since some cultivars, such as ‘Common Antonovka’ and F2-26829-2-2, and the accession of *M. micromalus*, were derived from different genetic backgrounds compared to the other accessions, and absence of sequence coverage in those regions could have been due to genomic variation. Finally, all SNPs with an allele frequency (AF) = 1 were discarded since this equated to all the re-sequenced varieties carrying an allele that was different from the ‘Golden Delicious’ reference genome. Such SNPs were discarded as they were likely to represent potential false SNPs resulting from sequencing errors in the ‘Golden Delicious’ reference sequence, or rare alleles derived from ‘Golden Delicious’. For the same reason, rare alleles derived from other cultivars were discarded during the SNP selection phase described below.

### Illumina Specific Filtering and SNP Selection

Quality filtered variants from the pipeline described above were then processed to meet Illumina Infinium II array design requirements (http://res.illumina.com/documents/products/technotes/technote_iselect_design.pdf). This third step removed variants that were either indels, A/T or C/G transversions, or tri-allelic SNPs. Additionally, variants located at sites containing additional high quality SNPs in either the up/down-stream 50 bp; or that contained both up-stream and down-stream sequence that appeared multiple times across the genome in the directly flanking 24 bp were removed [6]. Approximately one million SNPs obtained in this way were subsequently submitted to the Illumina Assay Design Tool (http://support.illumina.com/array/array_software/assay_design_tool.ilmn) for a preliminary estimation of the conversion rate (the Illumina SNP_Score). Following Illumina guidelines, markers with a SNP_Score below the threshold of 0.7 were discarded, while others were kept as high quality SNPs for the final selection of the 20,000 targets to be included on the 20****K Infinium array. In addition to these newly identified and validated SNPs from the discovery panel re-sequencing, 3,670 validated SNPs from the previously developed apple IRSC SNP WGG array were added to this selection. The SNPs selected from the IRSC array were chosen as they were robustly positioned in different genetic linkage maps, including those reported in [3] and [5].

**Figure 2:**
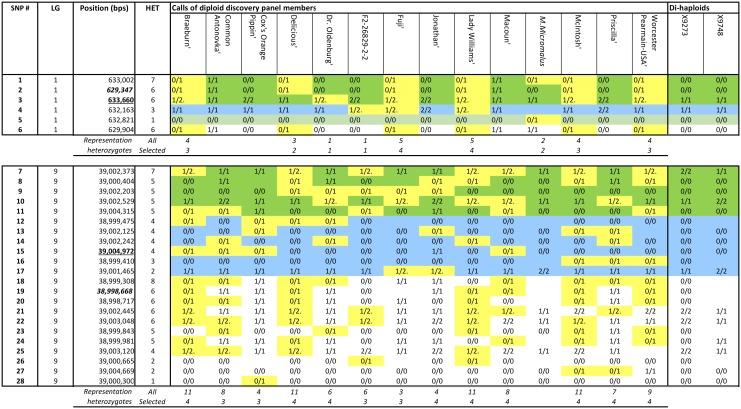
SNP selection within a single focal point (FP) of chromosomes (Chrs) 1 and 9. Genotype calls are presented for the discovery panel members: 0/1 and 1/2 indicates heterozygosity, all other codes indicate absence of polymorphism within an individual. Green, blue and orange filled cells relate to selected SNPs, having 5–8, 2–4, or just 1 heterozygous panel member/s, respectively. The physical positions at the extremities of the FP are in bold and italics (most proximal) or underlined (most distal). Within each FP, SNPs are sorted firstly by being selected or not, and secondly according to the number of heterozygous members (HET). In the Chr1-FP example, SNP-6 was not selected, as its heterozygosity pattern is identical to that of SNP-2. The single-member-heterozygote SNP-5 was included because no other more polymorphic SNP markers were available to reach the target of 11 SNPs and no nearby FPs had a specific SNP for the same panel member. In the Chr9-FP example all highly specific SNPs were ignored, as there were sufficient markers of higher heterozygosity available. The selected SNPs show a homogeneous representation of the diploid panel members.

The focal point strategy adopted for the design of this array followed that of the IRSC array design [3], with some modifications (Figure 2). The size of focal point intervals was reduced from 50****K bp, to 5****K bp up- or down-stream of the focal point itself to provide greater robustness in haplotype building (fewer recombination events within a focal point), and the number of SNPs within each FP was increased where feasible. Focal points evenly spread across the genome were first identified on the basis of the genetic positions available from previous mapping studies [3], [5], which resulted in a total of 718 FPs. A further 1,184 FPs were then added to cover physical intervals of an average of 400 Kbps to reach an average genetic distance of one FP per centiMorgan (cM) (using an expected Kbp/cM ratio of 440 Kbps based on a previous estimate by [2]). Finally, an additional 120 FPs were selected to enrich the ends of each of the 17 *Malus* chromosomes, making the number of FPs selected 2,022. Through a SNP short-listing process, up to 11 markers for each FP were selected. Entries featuring the same genotyping profile across the 14 re-sequenced accessions within a given FP were removed to avoid having too many SNPs at a single point potentially derived from the same haplotype. Additionally, SNPs displaying a percentage of heterozygous genotypes in the re-sequencing panel greater than 65% were also excluded as they were most likely to be the result of paralogy instead of true heterozygosity. In cases where 11 or fewer SNPs remained within a given FP, these were all selected for inclusion on the array. Where more than 11 SNPs remained, a step-wise inclusion procedure was followed. Firstly, up to five SNPs were selected that were polymorphic in 35–60% of the panel members, which generally corresponds to 5–8 members. Their heterozygous profiles had to be complementary as far as possible, and panel members had to be more or less equally represented, thus balancing the number of heterozygous SNPs across all (diploid) panel members. Secondly, this first set of highly polymorphic SNPs was complemented with SNPs that were polymorphic in 10–35% of the panel members, usually corresponding to 2–4 members, following the same principles on complementarity and representation. Thirdly, the remaining positions were filled up with SNPs that were polymorphic in just one panel member. This meant that complementarity within any given FP was accounted for as far as possible. This selection process was automated through a customized script that is available upon request from the corresponding author. Finally, the single member heterozygotes were manually scrutinized across all FPs, to ensure that FPs in the same region did not have a single heterozygous SNP for the same panel member. Also, *M. micromalus* specific SNPs were maintained at reduced frequency because of the currently limited use of this species in breeding (Figure 2).

### SNP Validation

The discovery panel was genotyped with the 20****K SNP array following the standard Illumina protocol detailed in the IRSC apple array and its application papers [3], [5]. In the framework of an ongoing PBA-based QTL mapping study of the FruitBreedomics project, over 1,600 seedlings from 21 full-sib families and their direct parents and additional progenitors were also genotyped and used for the construction of genetic maps. Although this work is not part of the current paper, we will present some of its outcomes, as they relate to the performance of the array. The full-sib families on which these data are based are listed in Table S1.

Genotyping data from the discovery panel and the full-sib families were analyzed using the GenomeStudio software (Illumina) with a GenCall threshold of 0.15 and a SNP filtering pipeline [23]. This employs the multipoint maximum likelihood mapping algorithm approach for cross pollinators in JoinMap 4.1 ([24], [25] E. Van de Weg, unpublished data).

Genotype calls from the 14 accessions of the discovery panel were compared with those obtained through high-coverage Illumina re-sequencing. Genotyping data were exported from GenomeStudio genotyping report with the option “Forward Strand” for consistency with the re-sequencing strand. Regarding re-sequencing data, only calls with a support of at least 10 reads were extracted from the .vcf file and used for the comparison.

## Results

### SNP Detection and Filtering

Sequencing resulted in 3,376 million reads from the 14 accessions (13 *M. × domestica* apple cultivars and one accession of *M. micromalus*), and an additional 809 million reads from the two DH accessions (http://bioinformatics.tecnoparco.org/fruitbreedomics/node/2). Table 1 details the depth of sequencing and the estimated genome coverage obtained for each of the 14 genotypes of the discovery panel. Variant calling resulted in a variant call format (.vcf) file containing 16,614,171 variants including indels, derived from all the re-sequenced accessions (Figure 1). A total of 662,559 variants were found to be heterozygous in one or both of the two DH accessions re-sequenced and were removed from further analysis. Thus 15,951,612 variants were retained after this filtering step. Following quality filtering described in the paragraph “Read Alignment, Variant Detection and Quality Filtering” of the Methods section, 13,373,299 variants were removed from further analysis, leaving 2,578,313 variants.

### SNP Selection

When quality filtered variants were processed to meet the Illumina Infinium II array requirements, a further 1,719,293 variants were removed, leaving 859,020 SNPs that were submitted to the Illumina Assay Design Tool pipeline. Following the Illumina recommendations and the parameters used by [3], 108,257 SNPs with SNP_Score below 0.7 were discarded leaving 750,763 high quality SNPs from the discovery panel for the selection of targets to be included on the array.

**Figure 3:**
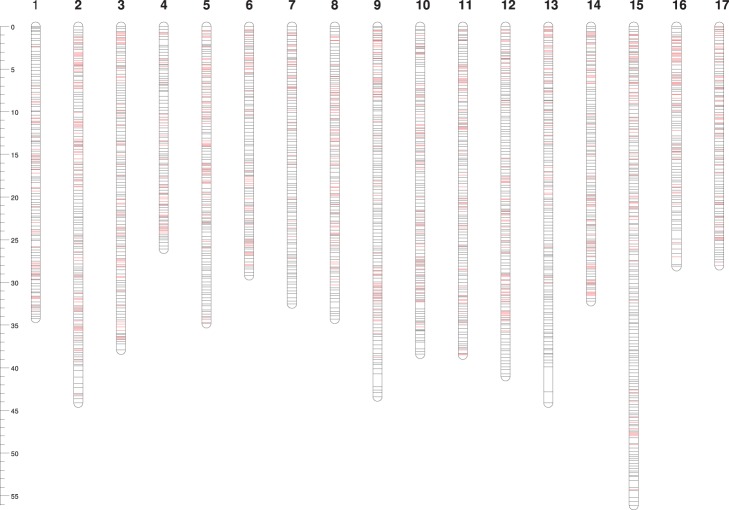
Distribution of focal points (FPs) (black lines) and IRSC SNPs (red lines) in the apple physical map v2. All positions are in Mbps. The average distance between FPs is 311 Kbps. Only two regions longer than 1 Mbps located at the distal end of Chrs 9 and 13 are not covered by FPs and SNPs from the 8****K IRSC SNP array. Scale bars = 5 Mbps.

Using the focal point approach detailed in the methods, 16,330 SNPs were identified from the re-sequencing of 14 genotypes of the discovery panel, as well as 3,670 validated markers from a previous IRSC array [3] and submitted to Illumina for array production. The Infinium array manufacturing produced a total of 18,019 SNP probes (14,714 newly identified SNPs and 3,305 from the IRSC array) to be incorporated in the final array. A total of 15,669 SNPs were located in 2,019 focal points (Figure 3). This included 955 SNPs from the IRSC array, which meant that some FPs contained more than 11 SNPs. The remaining 2,350 SNPs from the IRSC array did not fall in a FP and their physical position has been highlighted in Figure 3. The number of SNPs per FP ranged from 1 to 15, with a mean of 7.7. The mean distance between focal points was 311 Kbps, with two regions larger than 1 Mbps at the distal end of chromosomes 9 and 13. Overall, most regions that were not effectively covered by the IRSC array are now well represented by newly designed markers.

### SNP Mapping and Validation

Within the framework of the FruitBreedomics project, 21 full sib families were SNP genotyped, using the 20****K array, and were used for the generation of linkage maps. This resulted in the genetic mapping of 15.8****K SNP markers, and included 12,611 (success rate 86%) newly developed SNPs and 3,160 (success rate 96%) from the IRSC array that were informative in this new array and were mapped in at least one of the full-sib families screened (Table S2, Dataset S1). Of the non-mapped markers, 271 currently true monomorphic SNP markers may be mapped once screened over a wider germplasm set, as they are based on a single polymorphism in one of the more specific discovery panel members (e.g. *M. micromalus* or ‘Common Antonovka’). Additionally, 747 SNPs that featured complex cluster patterns (suggesting the probes annealed to paralogous genomic regions or contained additional SNPs within the probe binding sites) were found informative by visual inspection but would have required additional elaboration on the calling pipeline or manual annotation as previously reported in [6].

**Figure 4:**
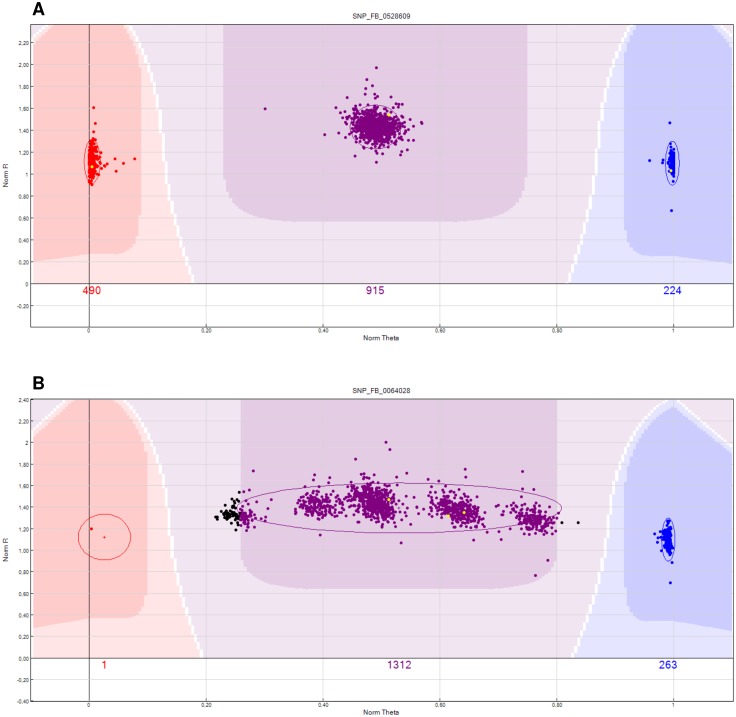
GenomeStudio cluster plot for the 21 F_1_ full-sib families used for the construction of genetic maps. Yellow dots indicate panel members that were re-sequenced and used as parents. Genotypes are called for each sample (dot) by their signal intensity (norm R) and Allele Frequency (Norm Theta) relative to canonical cluster positions (dark shading) for a given SNP marker (red = AA, purple = AB, blue = BB) a) GenomeStudio cluster plot of a newly developed robust SNP marker. b) GenomeStudio cluster plot of a failed/difficult to score SNP marker.

The number of SNPs successfully mapped for each of the 21 full-sib families varied from ∼5.2****K to ∼8.5****K. Examples of a GenomeStudio cluster for a robust mapped SNP and a failed/difficult to score SNP are shown in Figure 4.

### Genotype Call Comparison

The concordance of genotype calls made through re-sequencing with those obtained from genotyping with the 20****K array was assessed for the set of 15,771 validated SNPs. Excluding missing data, on average 12,347 comparisons were made for each of the 14 accessions (min: 10,402 for *M. micromalus*; max: 12,647 for ‘Fuji’) and the average concordance was 95%. In the majority of cases, the genotype call discordance identified was for low frequency SNPs observed in just one or two accessions of the discovery panel. This could have been the result of unreliable genotype calls in the re-sequencing analysis, owing to a lower read depth in those specific regions, or it might have been caused by unreliable genotype calls made in the array analysis owing to the presence of additional SNPs in probe binding sequences.

## Discussion

The outcome of our present work, a 20****K WGG array for *M. × domestica* using Illumina Infinium technology, comprising a total of 18,019 SNPs, has greatly increased the genotyping and analysis opportunities for apple researchers and breeders. High throughput array-based genotyping has revolutionized the study of genome-wide genetic variation, reducing costs and increasing the reliability and efficiency of data produced, as well as significantly reducing the time spent on genotyping itself. Medium or high density arrays are available for a range of other crop plant species, including cherry [26], grapevine [27], maize [28], peach [22], potato [29], soybean [30], tomato [31], sorghum [32], white spruce [33], alfalfa [34], and rice [35]. Of our 18,019 apple SNPs, 88% were validated in 21 full-sib families, with a further reservoir of a thousand of potentially informative SNPs if a wider germplasm base had been surveyed or if the remaining markers had been called manually. An efficiency rate of 88% corresponds well with efficiencies calculated for arrays developed for other plant species such as peach (84.3%; [22]), and is significantly higher than that of the previous *Malus* IRSC array (72%) [3]. Taking into account the polyploid origin of apple, the efficiency rate of 88% is the highest reached to date for a species that has undergone a recent whole genome duplication (WGD). This increased array efficiency was achieved through the implementation of a novel SNP detection and filtering pipeline in the design of the array, together with a new SNP calling pipeline for the use of the array [23] and the screening of a relatively large number of mapping populations. The new SNP calling pipeline successfully avoided the problem of extensive genome paralogy in apple [2], which caused calling difficulties using the IRSC apple array [6]. We employed a more stringent probe design by removing regions that showed multiple matches in the genome of the 24 nucleotides from either side of the SNP. Moreover, more stringent SNP filtering was achieved by including two DH accessions as discovery panel members and removing heterozygous SNPs that were identified in either of those genotypes during the SNP selection process. Finally, the high coverage re-sequencing strategy applied in this work allowed genotypes to be called for most panel members, and a minimum of ten reads per variant was imposed to successfully call a genotype. The comparison of sequenced reads for a potential variant site across multiple samples has the potential to differentiate systematic sequencing errors from real SNPs [36]. Such an approach permitted the detection of variant carriers without any need for complicated bioinformatics algorithms such as the one reported by [37], and reduced the number of false positive SNPs included in the array design. Comparison of read alignments across multiple samples also has the potential to filter out SNPs that are an artifact of inaccurate read alignments [36]. Moreover, it allowed the selection of sets of both more widely and more narrowly polymorphic markers that together represented the diversity of the discovery panel. This combination is expected to be useful for the tracing of markers (SNP haploblocks) along pedigrees for several successive generations. We did not include many SNPs of low heterozygosity (MAF) as they would have low probability of heterozygosity, and thus a low probability of being informative. As a result of these measures, the number of true monomorphic SNPs contained on the final array was very low, approximately 1% of the total. The use of this array to screen the FruitBreedomics families (Table S1) demonstrated the robust performance of the array, as the maps constructed had an average of 6.8****K SNPs uniformly distributed along the genome of each parent.

The final set of 2,578,313 high quality SNPs detected using the 14 re-sequenced accessions of the discovery panel corresponds to an average number of 4.8 SNPs/1,000 bp, in the 530 Mbps assembled and anchored ‘Golden Delicious’ reference genome sequence. This value was similar to the value reported in the apple genome paper [2] (4.4 SNPs/1,000 bp) but is somewhat higher than that found when surveying the polymorphism rate within a set of *M. × domestica* cultivars [38] (3.8 SNPs/1,000 bp), where analyses were limited to genic regions. Since, in contrast to what was done for the IRSC apple array [3], SNPs in this study were not selected exclusively from coding regions of the ‘Golden Delicious’ reference genome, the SNP heterozygosity rate reported here probably more closely represents the real genome-wide heterozygosity rate of *M. × domestica*. However, the actual SNP density may still be higher, as some of the filtered-out SNPs may actually be true polymorphisms.

Other methods have been developed recently for high-throughput genotyping of eukaryotic genomes using short-read sequencing technologies [39]–[41], and these techniques have been employed in the development of linkage maps and the identification of markers linked to agronomic traits in plants. Such studies include, for instance, genotyping by sequencing (GBS) based genetic maps of *Rubus idaeus* [42], *Hordeum vulgare* [43], [44], and restriction-site associated DNA (RAD) sequencing in *Lolium perenne* [41], *Hordeum vulgare* [45] and *Lupinus angustifolius* [46]. These techniques employ reduced genome representation achieved through restriction enzyme digestion and subsequent PCR analysis from adapted linker sequences, and require no *a priori* knowledge of the SNPs being interrogated, making them useful for genetic analysis in species where no reference sequence is currently available. In addition, since up to 96 samples can be multiplexed in a single lane of Illumina HiSeq sequencing, the genotyping cost per sample can be as low as USD12.00 at the time of writing (http://www.igd.cornell.edu/index.cfm/page/GBS/GBSpricing.htm). However, despite clear advantages to the use of GBS under certain experimental conditions for under-resourced species, GBS datasets contain a large proportion of missing values and false homozygote calls, due to low, and uneven genome coverage among individuals (36% in the case of *Rubus*; [42]), and hence data imputation strategies are required for effective data analysis. Moreover sampling chromosomes based on restriction digestion may introduce a bias in allele frequency estimation due to polymorphisms in restriction sites [47]. Whilst the cost of consumables for genotyping per individual is higher employing WGG arrays than for GBS, and only previously characterized SNPs that are present on the array can be interrogated, the data produced are robust and reliable, typically containing almost no missing values. Moreover when a robust and efficient SNP calling pipeline is available, there is less need for bioinformatic capacity, reducing the time and cost associated with data analysis. Coupled with the cost-effective production of arrays such as the one described in this investigation, containing nearly 16,000 validated SNPs, genetic analysis using WGG arrays provides tangible advantages over GBS, particularly for PBA, where genotyping is employed in related germplasm, and variation in hybridization efficiency and indel related null-alleles can be accounted for.

### Concluding remarks

We have developed a high-throughput WGG array for apple containing over 16****K validated SNP markers, spanning the apple genome at over 2,000 focal points evenly distributed throughout the 17 chromosomes of *M. × domestica*. Through the use of a novel SNP selection strategy informed through the design of the IRSC WGG array [3] and validation of data generated with that array [5], [6], [10], we have increased the robustness of the markers contained on the array, and reduced the occurrence of monomorphic SNPs and those that display cluster patterns indicating binding to paralogous loci or binding sites containing additional SNPs. The newly developed array, with its high density of polymorphic, validated SNPs, and its suitability for building multi-allelic SNP haploblocks, is expected to be of great utility for pedigree-based analysis, genomic selection and population genetics studies in *M. × domestica.* Moreover, the approach in SNP filtering and array design may be of use for the development of much higher density arrays for genome-wide association studies.

## Supporting Information

Table S1 **Full-sib families screened with the 20 K SNP array.** Parents and number of seedlings are listed. Pedigrees of the X-numbered accessions are reported by [11].
(DOCX)

Table S2 **List of the 18,019 SNPs included in the 20 K SNP array.** For each SNP, the physical location (Chromosome, Position), minor allele frequency (MAF), validation in 21 full-sib families, SNP genotype detected by the Illumina array, Illumina strand, source strand, source sequence, probe sequence, genotype calls (gt) made through re-sequencing and no. of high-quality bases (dp) for each discovery panel member, are reported.
(XLSX)

Dataset S1 **VCF file for the all newly discovered variants included in the 20 K SNP array.**
(ZIP)
