# Supplementary material for: Development and Validation of a 20K Single Nucleotide Polymorphism (SNP) Whole Genome Genotyping Array for Apple (Malus × domestica Borkh)
Source: PLoS One. 2014 Oct 10;9(10):e110377. doi: 10.1371/journal.pone.0110377 (PMC4193858; doi:10.1371/journal.pone.0110377)
Supplement: Table S1 — Full-sib families screened with the 20 K SNP array. Parents and number of seedlings are listed. Pedigrees of the X-numbered accessions are reported by [11]. (DOCX) [file pone.0110377.s001.docx]

**Table S1**. **Full-sib families screened with the 20K SNP array**. Parents and number of seedlings genotyped. Pedigrees of the X-numbered accessions are reported by [11].

| **Family** | **Mother** | **Father** | **No.of seedlings** |
| --- | --- | --- | --- |
| 12_B | ‘Generos’ | X6417 | 50 |
| 12_E | ‘Generos’ | X6683 | 58 |
| 12_F | X3318 | X6564 | 48 |
| 12_I | X3263 | X3259 | 47 |
| 12_J | X3318 | ‘Galarina’ | 24 |
| 12_K | X6679 | X6808 | 47 |
| 12_N | X3305 | X3259 | 48 |
| 12_P | ‘Rubinette’ | X3305 | 48 |
| DiPr | ‘Discovery’ | ‘Prima’ | 77 |
| DLO_12 | 1980-15-25 | 1973-1-41 | 225 |
| FuGa | ‘Fuji’ | ‘Gala’ | 141 |
| FuPi | ‘Fuji’ | ‘Pinova’ | 92 |
| GaPi | ‘Gala’ | ‘Pinova’ | 43 |
| I_BB | X6417 | X6564 | 43 |
| I_CC | X6679 | Dorianne | 50 |
| I_J | X3318 | X3263 | 48 |
| I_M | X6683 | X6681 | 46 |
| I_W | X6398 | X6683 | 45 |
| JoPr | ‘Jonathan’ | ‘Prima’ | 175 |
| PiRea | ‘Pinova’ | ‘Reanda’ | 46 |
| TeBr | ‘Telamon’ | ‘Braeburn | 202 |
| Total |  |  | 1603 |
